# Differential modulation of innate immune response by lipopolysaccharide of *Leptospira*

**DOI:** 10.1098/rsob.230101

**Published:** 2023-11-08

**Authors:** Vivek P. Varma, Ramudu Bankala, Ajay Kumar, Shashikant Gawai, Syed M. Faisal

**Affiliations:** ^1^ Laboratory of Vaccine Immunology, National Institute of Animal Biotechnology, Hyderabad 500032, India; ^2^ Graduate Studies, Manipal Academy of Higher Education, Manipal 576104, Karnataka, India; ^3^ Regional Centre for Biotechnology, Faridabad, India

**Keywords:** *Leptospira*, lipopolysaccharide, inflammasome, apoptosis, innate response

## Abstract

Leptospirosis is a worldwide zoonosis caused by pathogenic *Leptospira* spp. having more than 300 serovars. These serovars can infect a variety of hosts, some being asymptomatic carriers and others showing varied symptoms of mild to severe infection. Since lipopolysaccharide (LPS) is the major antigen which defines serovar specificity, this different course of infection may be attributed to a differential innate response against this antigen. Previous studies have shown that *Leptospira* LPS is less endotoxic. However, it is unclear whether there is a difference in the ability of LPS isolated from different serovars to modulate the innate response. In this study, we purified LPS from three widely prevalent pathogenic serovars, i.e. Icterohaemorrhagiae strain RGA, Pomona, Hardjo, and from non-pathogenic *L. biflexa* serovar semeranga strain Potac 1 collectively termed as L-LPS and tested their ability to modulate innate response in macrophages from both resistant (mice) and susceptible (human and bovine) hosts. L-LPS induced differential response being more proinflammatory in mouse and less proinflammatory in human and bovine macrophages but overall less immunostimulatory than *E. coli* LPS (E-LPS). Irrespective of serovar, this response was TLR2-dependent in humans, whereas TLR4-dependent/CD14-independent in mouse using MyD88 adapter and signalling through P38 and ERK-dependent MAP kinase pathway. L-LPS-activated macrophages were able to phagocytose *Leptospira* and this effect was significantly higher or more pronounced when the macrophages were stimulated with L-LPS from the corresponding serovar. L-LPS activated both canonical and non-canonical inflammasome, producing IL-1β without inducing pyroptosis. Further, L-LPS induced both TNF-mediated early and NO-mediated late apoptosis. Altogether, these results indicate that L-LPS induces a differential innate response that is quite distinct from that induced by E-LPS and may be attributed to the structural differences and its atypical nature.

## Graphical abstract

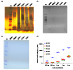

## 1. Introduction

Leptospirosis is a zoonotic disease caused by the Gram-negative bacterium *Leptospira* spp. [1]. It is prevalent in tropical areas, affecting more than one million people annually who are living in impoverished communities [2,3]. The epidemiology of the disease is dynamic and complex, with a wide variety of clinical manifestations ranging from a mild flu-like illness to severe infection leading to multi-organ dysfunction. The major challenge in combating this zoonosis has been the unavailability of early diagnostics and potent vaccines that can induce cross-protection against various serovars [4]. Understanding how *Leptospira* escapes from the host's innate immune defences to disseminate and colonize in multiple organs for establishing infection will aid in devising prophylactic strategies.

The host innate immune response is the first line of defence mediated by innate immune cells like dendritic cells, neutrophils and macrophages, which eliminate pathogens by a variety of mechanisms [5]. These cells recognize the pathogen-associated molecular patterns (PAMPs) such as lipopolysaccharide (LPS), surface proteins, glycolipids, carbohydrates, DNA or RNA through their specialized pattern recognition receptors (PRRs) such as Toll-like receptors (TLRs) leading to the activation of innate immune cells characterized by secretion of proinflammatory cytokines (IL-6, TNF-α, IL-12, IL-1β, IFN-α) via activation of mitogen-activated protein (MAP) kinase pathway and expression of surface molecules (CD80, CD86, MHC-II) which subsequently activate adaptive response [6,7]. The NOD-like receptors (NLRs) present in the cytosol are involved in the activation of the inflammasome, which is an essential arm of innate immunity [8]. Among them, NLR pyrin 3 (NLRP3) is the most common, which oligomerizes and binds apoptosis-associated speck-like protein containing a CARD (ASC) adaptors to form signalling platforms called canonical inflammasomes, which allows cleavage of caspase1, which in turn cleaves pro-IL-1β and IL-18 into mature cytokines to mediate potent inflammatory response [9,10]. Further, NLRP3 is also involved in the activation of non-canonical inflammasome involving activation of caspases 4/5 (human)/caspase 11 (mouse) and subsequent cleavage of GSDMD (gasdermin family protein), which oligomerizes to form pores on the plasma membrane, leading to inflammatory lytic cell death called pyroptosis [11–14].

Bacterial LPS is a potent activator of innate immune response and can cause the life-threatening syndrome of septic shock, which is the reason for naming LPS as an endotoxin [15]. LPS binds with the CD14/TLR4/MD2 receptor complex in many host cell types, such as monocytes, dendritic cells, macrophages, neutrophils and B cells, leading to activation and production of proinflammatory cytokines [16,17]. Stimulation with LPS can enhance the phagocytic ability of these innate immune cells [18,19]. Intracellularly, LPS can activate both canonical and non-canonical inflammasomes leading to the production of IL-1β for mediating inflammation or inducing pyroptotic cell death [20]. LPS can induce both TNF-α-mediated early or NO-mediated late apoptosis [21]. *Leptospira* LPS is a major antigen which is not only essential for the outer membrane integrity but also contributes to its virulence [22]. Leptospires have huge LPS biosynthesis loci of approximately 100 genes, all encoded on the same DNA strand [22]. The complete structure of LPS is unknown, as are the roles of individual proteins in LPS synthesis [23]. It is the molecular basis for its classification into more than 300 different serovars. LPS mutant is attenuated in virulence, and immune response against LPS can induce protection in an animal model [22]. *Leptospira* LPS is less endotoxic and atypical and differentially recognized through both TLR2 and TLR4 in humans [24]. Recent studies have demonstrated that *Leptospira* LPS can activate both canonical and non-canonical inflammasomes and can induce apoptosis without pyroptotic cell death [25]. Biochemical analysis of LPS from different pathogenic serovars (Autumnalis, Australis, Ballum, Grippotyphosa, Pomona) and the non-pathogenic serovar Andamana revealed significant structural differences in them; however, it is not known if LPS isolated from different pathogenic serovars differ in their ability in activating innate response in macrophages of both resistant (mice) and susceptible (human, bovine) hosts [26].

In the present study, we purified LPS from three widely prevalent pathogenic serovars, Icterohaemorrhagiae strain RGA (R-LPS), Pomona (P-LPS), Hardjo (H-LPS), and from non-pathogenic *L. biflexa* serovar semeranga strain Potac1 (S-LPS), collectively termed as *Leptospira* LPS (L-LPS), and tested their differential ability to activate macrophages from mouse, human and bovine host. Using cell-based assays, we then analysed the adapter molecule and signalling pathways involved in the activation of macrophages to understand if they are distinct from those involving *E. coli* LPS (E-LPS). We further assessed the differential ability of L-LPS in enhancing phagocytosis, activating inflammasome and inducing apoptosis or pyroptosis in these macrophages.

## 2. Results

### 2.1. Purification and characterization of L-LPS

**Figure 1.**
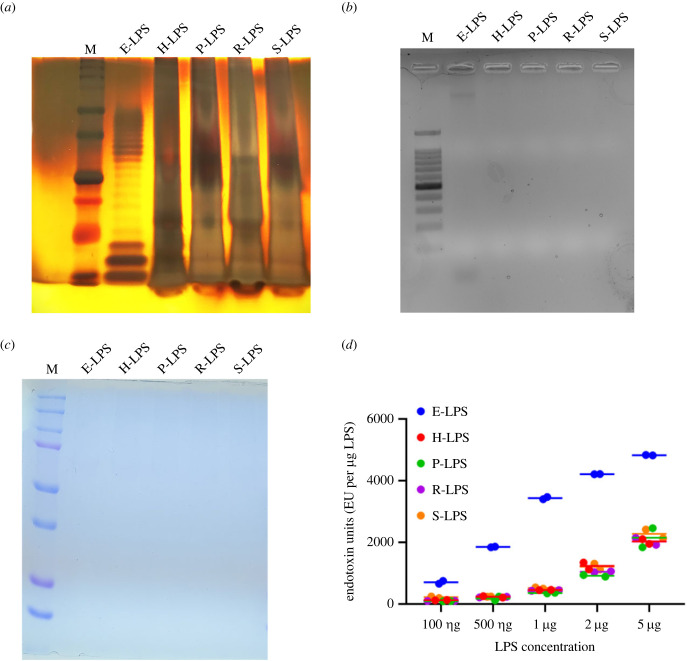
Purification and characterization of *Leptospira* LPS from different serovars. (*a*) PAS staining of L-LPS. LPS from different serovars were purified using a modified hot water/phenol method, and 5 µg of each was subjected to SDS-PAGE followed by (periodic acid silver) PAS staining as described in the Material and methods. Lane M: protein marker (BIO-RAD); E-LPS: commercially procured *E. coli* LPS 0111:B4; H-/P-/R-/S-LPS are LPS isolated from *Leptospira* Hardjoprajitno, Pomona, RGA and SSP, respectively. (*b*) Analysis of protein contamination in purified L-LPS. 30 µg of each E-LPS and L-LPS is loaded onto SDS-PAGE gel and run until the gel front reaches the end and is visualized using coomassie brilliant blue (CBB) R250, followed by destaining. Lane M represents the protein marker (BIO-RAD). (*c*) Analysis of DNA contamination in purified L-LPS. 30 µg of each E-LPS and L-LPS is loaded onto an agarose gel and run until the gel front reaches the end and is visualized using EtBr-agarose gel electrophoresis. Lane M represents the 100 bp DNA ladder (NEB). (*d*) Evaluation of endotoxicity of purified L-LPS. Purified L-LPS were tested for endotoxicity in varying concentrations (100 ng, 500 ng, 1 µg, 2 µg, 5 µg) using a commercial colorimetric LAL assay kit following the manufacture's protocol. The endotoxicity was compared with commercial E-LPS. The data are presented as endotoxin units per microgram LPS.

We purified LPS from three pathogenic serovars (*L. interrogans* serovar Icterohaemorrhagiae strain RGA, serovar Pomona, serovar Hardjo) and one non-pathogenic serovar (*L. biflexa* serovar semeranga strain Potac 1). The purified LPS samples were visualized by staining in periodic acid silver staining. Our results show that in contrast to E-LPS, which had a characteristic ladder pattern (a typical feature of Gram-negative LPS), L-LPS showed a smear-like appearance with fast-moving low molecular weight (MW) O-antigen of different lengths. The banding pattern also varied among serovars, where Hardjo and Pomona showed low-MW bands, and RGA and Patoc1 showed higher MW, forming a smear-like pattern (figure 1*a*). Lipid A-core of LPS migrated very near the dye-front and stained very intensely appearing as a black region at the bottom of the gel. To check the DNA contamination in isolated LPS, we ran on an agarose gel stained with ethidium bromide, and our result shows a lack of any band in L-LPS. However, a mild band was visible in commercially procured *E. coli* LPS (figure 1*c*). To rule out the protein contamination, a primary immune activator, LPS samples, were subjected to SDS-PAGE and stained with coomassie blue, and our result shows no visual bands, indicating the absence of proteins (figure 1*c*) in the L-LPS preparation. The endotoxin levels in isolated LPS were tested by LAL assay, and our result showed that the levels increased with increasing concentration of both E-LPS and L-LPS; however, the endotoxin levels in L-LPS were significantly lower than those in E-LPS (figure 1*d*).

### 2.2. L-LPS induced a differential innate response in various host macrophages

**Figure 2.**
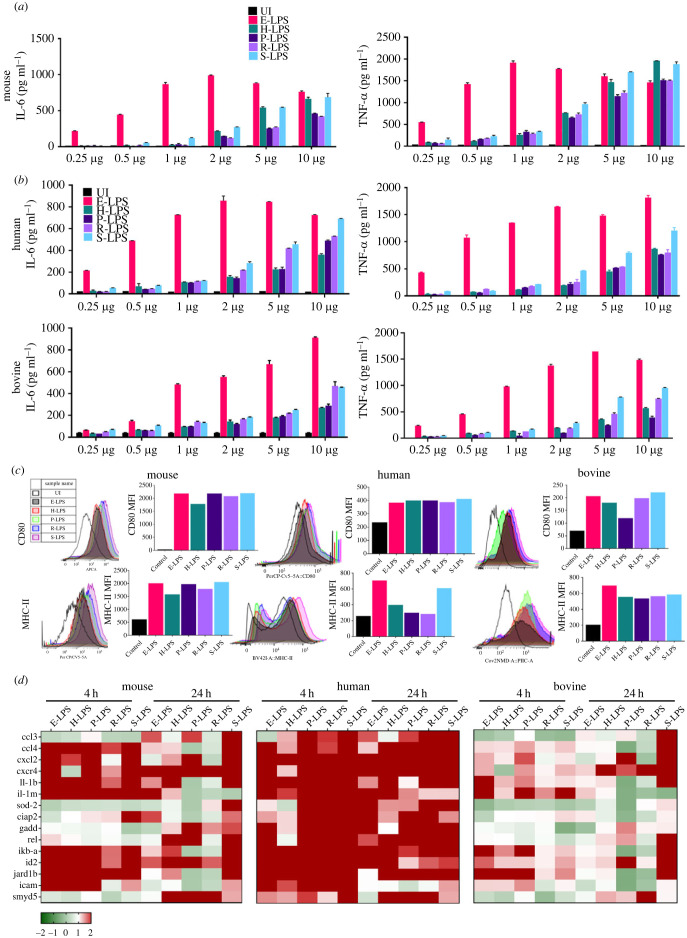
L-LPS induces differential innate responses in various host macrophages. (*a* & *b*) L-LPS induces proinflammatory response in mouse, human and bovine macrophages. RAW264.7 cells, PMA-treated THP1 cells and BoMac cells were stimulated with 500 ng ml^−1^ of *E. coli* LPS or 0.25–10 μg ml^−1^ of L-LPS for 24 h as described in materials and methods. The supernatants were collected to measure the IL-6 and TNF-α using R&D sandwich ELISA kit as per the manufacturer instructions. (*c*) L-LPS enhances the expression of surface markers in mouse, human and bovine macrophages. RAW264.7 or THP1 (PMA-treated) or BoMac cells were stimulated with *E. coli* LPS (500 ng ml^−1^) or L-LPS (2 µg ml^−1^) for 24 h at 37°C in the presence of 5%CO_2_. Cells were harvested and stained with fluorochrome-conjugated host-specific monoclonal antibodies against CD80 and MHC-II, and data were acquired by flow cytometry and analysed by FlowJo as mentioned in the Material and methods. (*d*) RT-PCR analysis of expression of genes related to innate response and cell physiology in various host macrophages stimulated with L-LPS. RAW264.7 or THP1 (PMA-treated) or BoMac cells were stimulated with *E. coli* LPS (500 ng ml^−1^) or L-LPS (2 µg ml^−1^) for 4 h or 24 h at 37°C/5% CO_2_ and then harvested to isolate total RNA. RNA was converted to cDNA, and modulation of select genes was analysed by qRT-PCR as described in the Material and methods. The data were presented as fold changes between stimulated cells versus control and normalized to GAPDH.

To check if there is a difference in innate immune activity by LPS isolated from three different pathogenic serovars and one non-pathogenic serovar, we stimulated mouse macrophages with L-LPS and analysed the production of proinflammatory cytokines (IL-6 and TNF-α). Our result shows that L-LPS from various serovars induced activation of macrophages in a dose-dependent manner; however, it was less stimulatory as compared to E-LPS, as evident from the production of proinflammatory cytokines (IL-6, TNF-α). While E-LPS induced a significant level of cytokines at a dose of 0.5 µg ml^−1^, L-LPS induced a similar response at a dose of 2–5 µg ml^−1^ (figure 1*a*). Of various LPS serovars tested, H-LPS and S-LPS induced significantly higher level of cytokines. The results were consistent in primary cells (bone marrow-derived dendritic cells) stimulated with L-LPS (electronic supplementary material, figure S1*a*). Further, L-LPS induced significant levels of other proinflammatory cytokines like IL-12 and IL-23; however, their levels were lower than those induced by E-LPS (electronic supplementary material, figure S1*b*). Since LPS is known to induce the production of the anti-inflammatory cytokine IL-10 we analysed its levels, and our result shows that L-LPS from pathogenic serovars induced significantly higher levels of IL-10 than S-LPS (electronic supplementary material, figure S1*c*). To check if the innate response is similar in susceptible host cells, we stimulated human (THP1) and bovine (BoMac) macrophage cell lines with L-LPS. Our result shows that L-LPS induced a dose-dependent activation of both human and bovine macrophages; however, it Induced a significantly lower level of cytokines as compared to mouse macrophages (figure 2*b*). To check if the activated cells upregulated the expression of costimulatory molecules (CD80, CD86, CD40) and maturation marker (MHC-II), we analysed their expression by flow cytometry. Our result shows that in mouse macrophages (figure 2*c* and electronic supplementary material, figure S1*d*), L-LPS-induced expression of these molecules was similar to the level induced by E-LPS; however, their expression level was lower in human and bovine macrophages (figure 2*c* and electronic supplementary material, figure S1*e*). Further, S-LPS induced activation of macrophages similar to E-LPS; however, LPS from pathogenic serovars induced a lower level of activation. To check if L-LPS modulated cell physiology and expression of other innate immune genes, we analysed the expression of cytokines, chemokines, apoptosis, stress, transcriptional and growth-promoting genes in L-LPS-stimulated macrophages. Our result shows that L-LPS modulated the expression of several cytokines (il-1b and il-1rn), chemokines (ccl4, cxcl2, and cxcr4), stress response (sod2), apoptosis (ciap2 and gadd), transcription (ikb-a), cell cycle and growth (id-2, jard1b) marker related genes at 4 and 24 h in all host macrophages (figure 2*d*). Taken together, these results indicate that L-LPS induced strong activation of macrophages from the resistant host (mice); however, it was less stimulatory in macrophages from susceptible hosts (human and bovine).

### 2.3. L-LPS is atypical, which signals through TLR2 to induce activation of human macrophages

**Figure 3.**
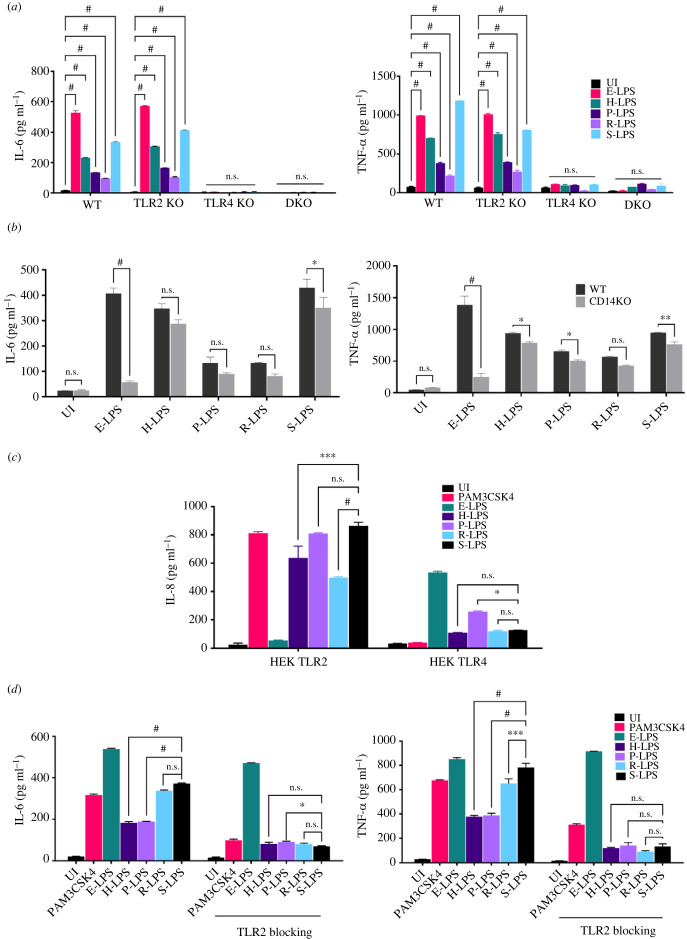
L-LPS induces TLR4-dependent activation in mouse, whereas TLR2-mediated activation in human macrophages. (*a*) Proinflammatory response of mouse macrophages stimulated with L-LPS. WT, TLR2KO, TLR4KO and DKO macrophage cell lines were stimulated with 500 ng ml^−1^ of *E. coli*, or 2 µg ml^−1^ of L-LPS for 24 h, and the supernatant was collected to measure IL-6 and TNF-α using R&D sandwich ELISA kit as per the manufacturer instructions. (*b*) Analysis of CD14-dependent activation of mouse macrophages stimulated with L-LPS. WT and CD14KO mouse macrophage cell lines were treated with 500 ng ml^−1^ of *E. coli* or 2 µg ml^−1^ of L-LPS for 24 h. The supernatant was collected to measure IL-6 and TNF-α using an R&D sandwich ELISA kit as per the manufacturer's instructions. (*c*) Analysis of IL-8 in HEK-hTLR2 and HEK-hTLR4 cells stimulated with L-LPS. HEK293T cells were transfected with hTLR2, hTLR4, MD2 and NF-kB reporter plasmids followed by stimulation with E-LPS (500 ng ml^−1^) or PAM3CSK4 (20 ng ml^−1^; TLR2 ligand) or L-LPS (2 µg ml^−1^) for 24 h and IL-8 was measured in the culture supernatant by sandwich ELISA kit. (*d*) Analysis of cytokines in THP1 macrophages pre-blocked with TLR2 monoclonal antibody followed by stimulation with L-LPS. THP1 macrophages were blocked with TLR2-specific monoclonal antibody for 2 h, followed by stimulation with E-LPS (500 ng ml^−1^) or PAM3CSK4 (20 ng ml^−1^) or L-LPS (2 µg ml^−1^) for 24 h at 37°C in the presence of 5% CO_2_ and cytokine levels of IL-6 and TNF-α were estimated in the culture supernatant by ELISA. Significant differences were calculated using one- or two-way ANOVA (^#^, ***, **, *, and n.s. indicate *p* < 0.0001, *p* < 0.001, *p* < 0.01, *p* < 0.05 and non-significant, respectively).

LPS is a known ligand of TLR4. Since leptospiral LPS is atypical and showed less endotoxicity, we tested the receptor involved in signalling. Murine WT, TLR2−/− (TLR2KO), TLR4−/− (TLR4KO) and TLR2−/−/4−/− (TLR2&4 DKO) macrophage cell lines were stimulated with L-LPS, and production of proinflammatory cytokines was analysed. Our result shows that TLR2KO macrophages produced a significant level of IL-6 and TNF-α albeit with some differences in their levels depending on stimulation with specific serovar LPS, whereas TLR4KO and DKO macrophages failed to induce these cytokines, indicating that like E-LPS, L-LPS also induces activation of mouse macrophages via signalling through TLR4 (figure 3*a*). To evaluate the role of CD14 in L-LPS mediated activation, we stimulated CD14KO macrophages with L-LPS and our result shows that in contrast to E-LPS which induced significantly reduced levels of cytokines, L-LPS induced cytokine levels similar to WT, indicating that CD14 does not play any significant role in L-LPS mediated TLR-4 signalling (figure 3*b*). Since previous studies have shown a role of TLR2 in L-LPS mediated signalling in human macrophages, we confirmed this by stimulating HEK cells expressing hTLR2 (HEK-TLR2) and hTLR4 (HEK-TLR4) with highly pure L-LPS followed by estimation of IL-8. Our result shows that while L-LPS stimulated, HEK-TLR2 produced significant levels of IL-8, HEK-TLR4 failed to produce significant levels of cytokines indicating that L-LPS binds to human TLR2 receptor instead of TLR4 to induce a proinflammatory response (figure 3*c*). We further confirmed this by stimulating THP-1 macrophages blocked with monoclonal antibodies against the TLR2 receptor. Our result showed that blocking the TLR2 receptor significantly reduced the ability of L-LPS-stimulated macrophages to produce cytokines; however, it did not have any effect on cells stimulated with E-LPS (figure 3*d*). Taken together, these results indicate that irrespective of serovar, L-LPS mediates TLR4-dependent/CD14-independent signalling in mouse whereas it signals through TLR2 in humans.

### 2.4. L-LPS induces activation of macrophages via P38 and JNK mediators of the MAP kinase pathway

**Figure 4.**
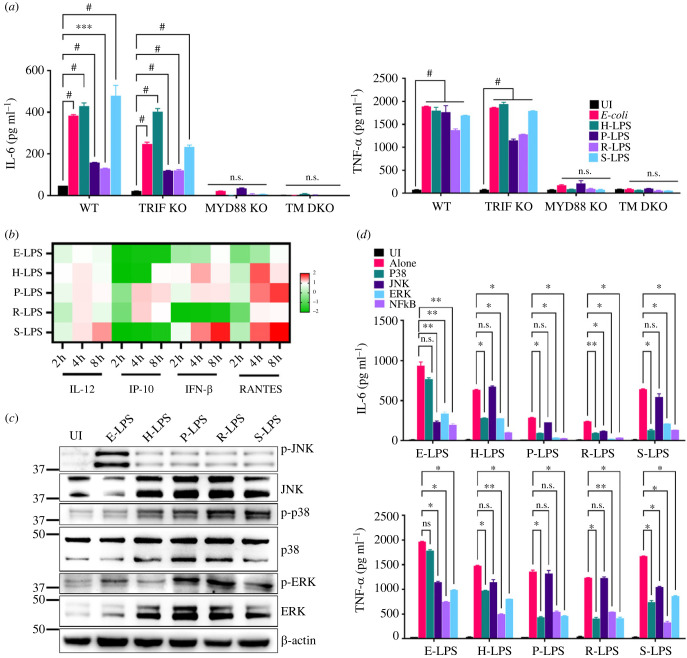
L-LPS induces the proinflammatory cytokines via the ERK and P38-dependent MAPK pathway signalling. (*a*) Analysis of adapter molecule involved in signalling through L-LPS. WT, MyD88 KO, TRIF KO and DKO (TRIF & MyD88 double knock out) mouse macrophage cell lines were treated with E-LPS (500 ng ml^−1^) or L-LPS (2 µg ml^−1^) for 24 h at 37°C in the presence of 5% CO_2_ and levels of IL-6 and TNF-α were measured in the culture supernatant using sandwich ELISA kit. (*b*) RT-PCR analysis of TRIF signalling associated cytokines and chemokines in L-LPS-stimulated macrophages. RAW264.7 cells were stimulated with E-LPS (500 ng ml^−1^) or L-LPS (2 µg ml^−1^) for 2 or 4 or 8 h at 37°C/5% CO_2_. Cells were recovered, and RNA was isolated and converted to cDNA, and IFN-1 pathway gene expression was analysed by qRT-PCR as described in the Material and methods. (*c*) Western blot analysis of phosphorylation of mediators of MAP kinase pathway in macrophages stimulated with L-LPS. RAW264.7 cells were stimulated with E-LPS (500 ng ml^−1^) or L-LPS (2 µg ml^−1^) for 24 h at 37°C in 5% CO_2_. Levels of P38, JNK and ERK1/2 and respective phosphorylated forms were analysed by western blot as described in Material and methods. (*d*) Analysis of MAP kinase signalling pathway in macrophages stimulated with L-LPS. RAW264.7 cells pre-treated with pharmacological inhibitors of NF-kB (SN50; 20 µM), JNK (SP600125; 40 µM) or P38 (SB203580; 30 µM) or ERK (U0126; 50 µM) for 30–60 min and then stimulated with E-LPS (500 ng ml^−1^) or L-LPS (2 µg ml^−1^) for 24 h at 37°C in the presence of 5% CO_2_ and supernatant was collected to measure levels of IL-6 and TNF-α by ELISA. Significant differences were calculated using one- or two-way ANOVA (^#^, ***, **, * and n.s. indicate *p* < 0.0001, *p* < 0.001, *p* < 0.01, *p* < 0.05 and non-significant, respectively).

To check if L-LPS induces signalling via membrane activation involving the TIRAP-MyD88 adapter or by endocytosis involving the TRAM-TRIF adapter, we analysed cytokines in MyD88 and TRIF-KO macrophages stimulated with L-LPS. Our result shows that while TRIF-KO macrophages induced significant levels of cytokines, MyD88-KO macrophages failed to induce these cytokines irrespective of which serovar L-LPS is used, indicating that L-LPS is primarily involving the MyD88 adapter for signalling (figure 4*a*). P-LPS and R-LPS treatment consistently showed low proinflammatory cytokine response, and we suspected the involvement of TRIF–TRAM adaptor molecule recruitment for the TLR4 activation. To evaluate TRIF–TRAM-dependent signalling, we stimulated mouse macrophages with L-LPS and analysed relevant IFN-1 cytokines (il-12, ip-10, ifn-β and rantes) at various time points by RT-PCR. Our result shows that P-LPS and R-LPS significantly upregulated the expression of rantes whereas H-LPS enhanced the expression of ifn-β (figure 4*b*). To analyse the MAP kinase pathway involved in signalling and subsequent production of cytokines, we stimulated mouse macrophages with L-LPS and analysed the phosphorylation of P38, JNK and ERK by western blot. Our results show that L-LPS induced strong phosphorylation of P38 and ERK, whereas E-LPS induced phosphorylation of JNK and ERK (figure 4*c*). Next, to elucidate the functional role of these kinases in L-LPS-induced macrophage activation and maturation, we used pharmacological inhibitors of these pathways and analysed cytokines in RAW264.7 cells pre-treated with or without inhibitors of NF-kB or JNK or p-38 or ERK. IL-6 and TNF-α production was significantly blocked (*p* < 0.05, 50% inhibition) by P38 inhibitor and by ERK and NF-kB inhibitors (*p* < 0.05, 30% inhibition) in macrophages stimulated with L-LPS (figure 4*d*). JNK inhibitor did not affect the production of cytokine, indicating that this pathway is not involved in signalling upon stimulation with L-LPS (figure 4*d*). All these results suggest that L-LPS primarily signals through MyD88 except P-LPS which involved a TRIF adapter also and stimulates the production of proinflammatory cytokines through P38, ERK and NF-kB pathways which is distinct from E-LPS.

### 2.5. L-LPS enhanced serovar-specific phagocytosis

**Figure 5.**
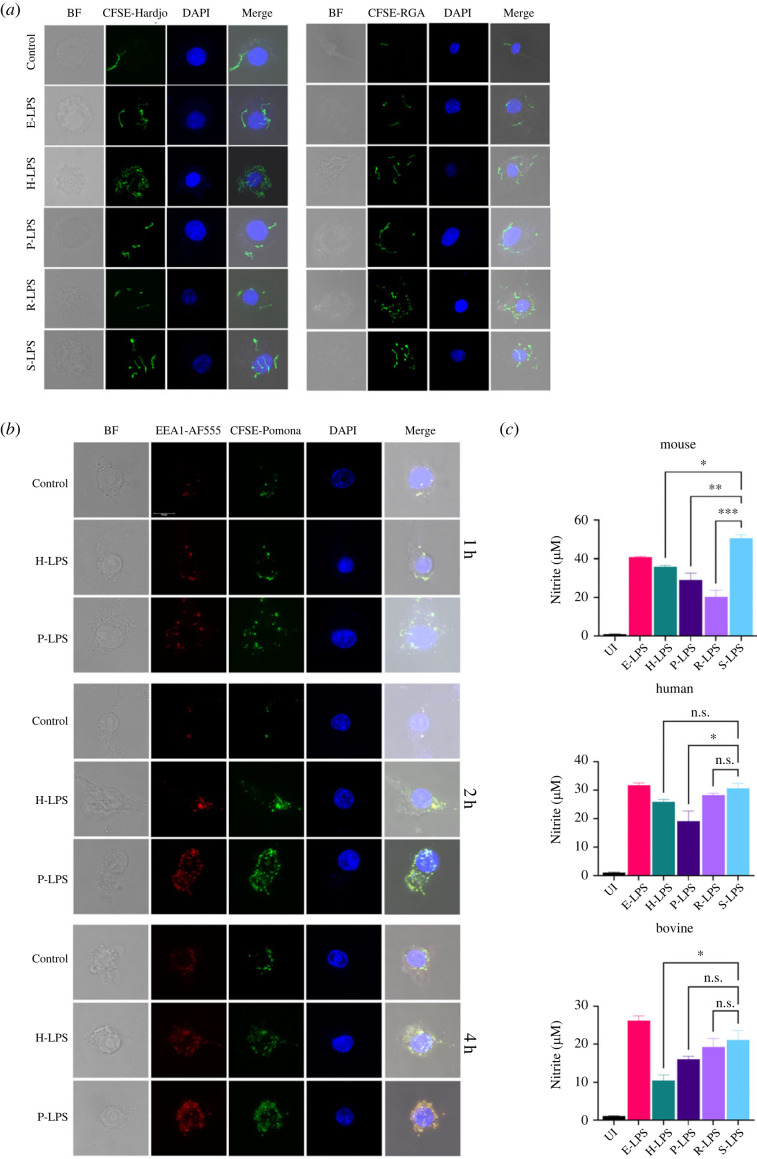
L-LPS enhances macrophage phagocytosis in a serovar-specific manner. (*a*) Confocal microscopy analysis of the uptake of *Leptospira* in macrophages stimulated with L-LPS. RAW264.7 cells were stimulated with E-LPS (500 ng ml^−1^) or L-LPS (2 µg ml^−1^) for 4 h followed by infection with CFSE-labelled Hardjo or RGA. Post 30 min of infection, cells were washed, fixed, mounted onto the slides with Vectashield mounting media containing DAPI, and examined under a ×63 confocal microscope using *z*-scanning as described in Material and methods. (*b*) Confocal microscopy analysis of phagocytosis of *Leptospira* in macrophages stimulated with L-LPS. RAW264.7 cells were stimulated with E-LPS or L-LPS for 4 h, followed by infection with CFSE-labelled Pomona for 90 min. After killing the extracellular bacteria, cells were fixed and stained with anti-EEA1 antibody, followed by Alexa Fluor 555 labelled 2° antibody staining. The cover slips were washed, mounted onto the slides and examined under a ×63 confocal microscope using z-scanning as described in the Material and methods. (*c*) Estimation of NO production in LPS-stimulated macrophages. Mouse (RAW264.7), human (THP1; PMA-treated) and bovine (BoMac) macrophage cells were stimulated with E-LPS (500 ng ml^−1^) or L-LPS (2 µg ml^−1^) at 37°C in 5% CO_2_. Nitric oxide (NO) level was estimated in the culture supernatant post-24 h of induction by Griess reagent. Concentration was measured from the standard graph as mentioned in the Material and methods.

It is known that the phagocytic activity of macrophages is augmented upon LPS treatment. To check if there is any difference in the ability of LPS isolated from different serovars in augmenting the uptake and subsequent phagocytosis, we analysed E-LPS- or L-LPS-stimulated mouse macrophages incubated with CFSE labelled *Leptospira* (Hardjo or RGA) by confocal microscopy. Our result shows that L-LPS stimulation enhanced the uptake of bacteria by macrophages; however, uptake of specific serovar (Hardjo) was significantly higher in macrophages if stimulated with its own LPS (H-LPS) (figure 5*a*). To confirm this serovar specificity, we incubated L-LPS-stimulated macrophages with different serovar (RGA) and our result shows that uptake was significantly higher in R-LPS-stimulated macrophages (figure 5*a*). To confirm that the increased uptake is correlated to enhanced phagocytosis, we analysed the co-localization of CFSE labelled *L. Pomona* in endosome using early endosome marker EEA-1. Our result shows that a significantly higher number of bacteria (*L. Pomona*) were localized as early as 1 h in the endosome of macrophages stimulated with P-LPS than those stimulated with H-LPS (figure 5*b*). A similar trend was observed at 2 h and 4 h. Since nitric oxide (NO) is a crucial player in phagocytosis, we evaluated its level in LPS-stimulated macrophages. Our result shows that levels of NO were significantly higher in macrophages stimulated with S-LPS (non-pathogenic) than any of the L-LPS from pathogenic serovars (figure 5*c*). R-LPS induced significantly lower levels of NO (figure 5*c*). Altogether, these results indicate that L-LPS can stimulate macrophages and enhance the uptake and subsequent phagocytosis of *Leptospira,* which is serovar-specific.

### 2.6. L-LPS activates both canonical and non-canonical inflammaosomes

**Figure 6.**
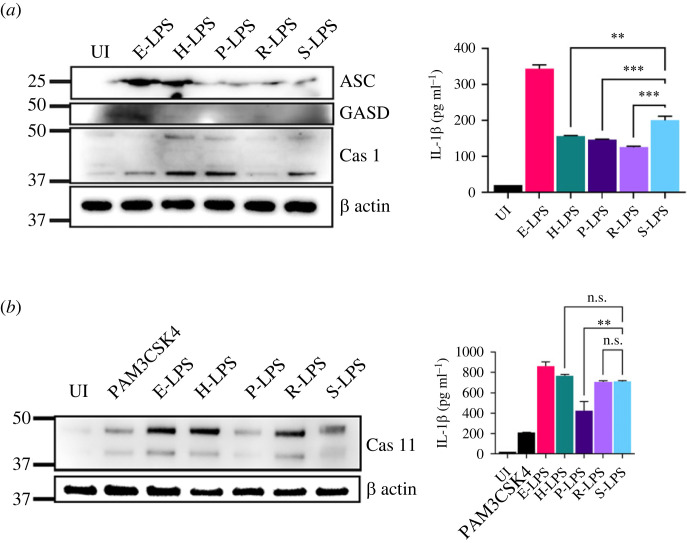
L-LPS induces activation of both canonical and non-canonical inflammasome without induction of pyroptosis. (*a*) Analysis of expression of caspase-1, ASC and GSMD by western blot and levels of IL-1β by ELISA. RAW264.7 cells were unstimulated (control) or stimulated with E-LPS (500 ng ml^−1^) or L-LPS (5 µg ml^−1^) for 24 h at 37°C in 5% CO_2_. Post-induction cells were washed with PBS and lysed with RIPA buffer. Levels of ASC, caspase-1 and gasdermin-d were analysed by western blot using specific antibodies as described in Material and methods. IL-1β in the culture supernatant was analysed by cytokine ELISA kit. Cell alone served as a negative control. (*b*) Analysis of expression of Cas11 by western blot and IL-1β in culture supernatant by ELISA. RAW264.7 cells were primed with PAM3CSK4 for 2 h and transfected with E-LPS (2 µg) or L-LPS (5 µg). Five hours after post-transfections, the cells were lysed and subjected to immunoblotting, followed by immunoprobing using an anti-mouse Cas11 antibody. The culture supernatant was used to measure the cytokine IL-1β levels. Cells alone or primed with PAM3CSK4 with transfection reagent served as a control.

Previous studies have demonstrated that *Leptospira* can activate inflammasome [27]. This activation is attributed to lipoproteins, to glycoproteins, and, more recently, to LPS [28]. To check if there is any differential activation of inflammasome in macrophages stimulated with LPS from different pathogenic serovars, we stimulated mouse macrophages with E-LPS and L-LPS. Our result shows that similar to E-LPS, L-LPS can activate canonical inflammasome characterized by expression of Cas1, ASC and subsequent release of IL-1β (figure 6*a*). Although *Leptospira* is not a typical intracellular bacterium, it is highly invasive and has been shown to enter and exit macrophages. Hence, it is likely that it might activate non-canonical inflammasome through its LPS and induce pyroptotic cell death. To evaluate the ability of L-LPS to activate non-canonical inflammasome, we primed the cells with PAM3CSK4 and then transfected L-LPS in mouse macrophages and analysed Cas11. L-LPS upregulated expression of Cas11 and subsequent activity correlating to the production of IL-1β (figure 6*b*). To analyse the Cas1/Cas11 mediated pyroptotic pathway, we assessed the cleavage of GSMD by western blot. Our result shows that in contrast to E-LPS, none of the L-LPS induced cleavages of GSMD as the cleaved fragment was not detected by a specific antibody (figure 6*a*). Overall, these results indicate that irrespective of the serovar from which it is isolated, L-LPS can induce activation of both canonical and non-canonical inflammasome to induce IL-1β but prevents induction of pyroptosis, thus preventing cell death.

### 2.7. L-LPS induces both TNF-α-mediated early and NO-mediated late apoptosis

**Figure 7.**
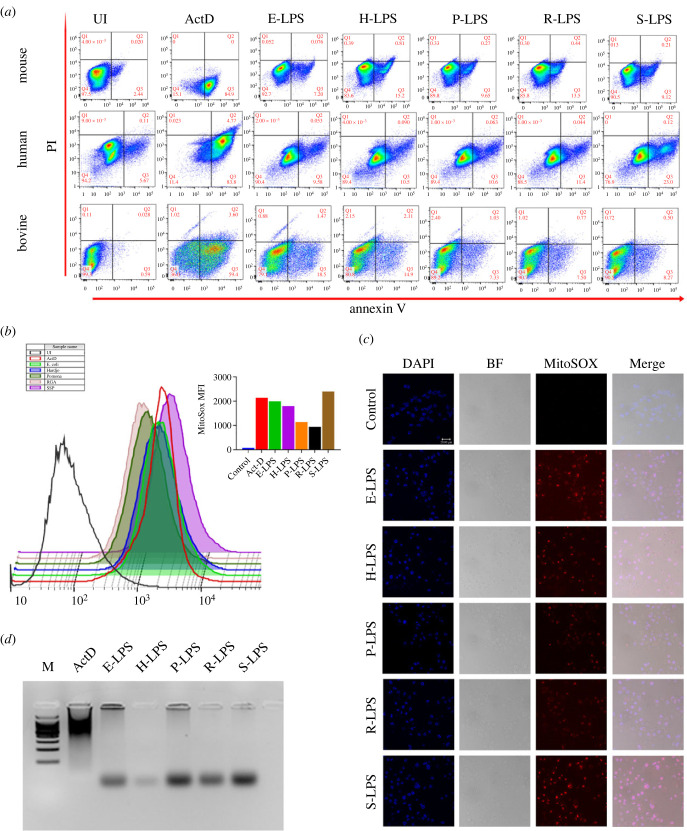
L-LPS induces apoptosis in macrophages of various hosts. (*a*) Estimation of TNF-α-mediated early apoptosis. Mouse (RAW264.7), human (THP1) and bovine (BoMac) macrophages were stimulated with ActD (100 ng ml^−1^; known apoptosis inducer) or 500 ng of *E. coli*, or 5 µg ml^−1^ of L-LPS for 24 h and stained with Annexin-V-FITC: PI apoptosis kit and analysed for apoptosis using flow cytometry. (*b*) Analysis of NO-mediated late apoptosis by L-LPS. RAW264.7 cells were stimulated with ActD (100 ng ml^−1^) or E-LPS (500 ng ml^−1^) or L-LPS (2 µg ml^−1^) for 24 h at 37°C in 5% CO_2_. Cells were harvested and stained with MitoSox red for 30 min as per the manufacturer's instructions, followed by washing, fixation, and analysis by flow cytometry using the PE channel. (*c*) Analysis of mitochondrial ROS production by confocal microscopy. RAW264.7 cells seeded on the coverslips were stimulated with ActD (100 ng ml^−1^) or E-LPS (500 ng ml^−1^) or L-LPS (2 µg ml^−1^) for 24 h at 37°C in 5% CO_2_. Cells were washed, fixed with 4% paraformaldehyde for 15 min, stained with MitoSox red for 30 min, washed thrice and mounted onto the slides with a mounting medium containing DAPI. Imaging was done at ×63 magnification with confocal microscopy. (*d*) Analysis of shearing of DNA in L-LPS-stimulated macrophages. RAW264.7 cells were stimulated as described above and then washed with PBS, lysed and centrifuged to precipitate the genomic DNA. Low molecular weight supernatant DNA was phenol-extracted twice and precipitated with 95% ethanol. The pellet was resuspended in Tris-EDTA buffer, and RNA was removed with RNase and analysed by electrophoresis in a 2% agarose gel containing ethidium bromide. Lane M represents the 100 bp DNA ladder.

To evaluate the role of L-LPS in inducing TNF-α-mediated apoptosis, we induced mouse, human, and bone macrophages with E-LPS and L-LPS and analysed the expression of phosphatidyl serine (PS) binding with Annexin-V antibody by flow cytometry. Similar to E-LPS, L-LPS induced a significant level of early apoptosis as measured by PS-positive cells (figure 7*a*). Among various pathogenic serovars, H-LPS induced higher apoptosis in mouse and bovine macrophages (15.2% and 14.9%), whereas non-pathogenic S-LPS induced a high level of apoptosis in human macrophages (23%) (figure 7*a*). To measure NO-mediated late apoptosis, we stimulated mouse macrophages with L-LPS and measured mitochondrial ROS using MitoSOX red dye. Our result shows that non-pathogenic S-LPS induced the highest amount of ROS production (figure 7*b*). Among LPS from pathogenic serovars, H-LPS induced a significantly high level of ROS comparable to the level induced by E-LPS estimated by flow cytometry (figure 7*b*). We also confirmed the mitochondrial ROS production by confocal microscopy. Confocal microscopic imaging demonstrated a significant increase in mitochondrial fluorescence intensity of MitoSOX red in L-LPS-treated cells (figure 7*c*). Since ROS can mediate DNA damage leading to programmed cell death, we analysed the shearing of DNA in L-LPS-treated cells, and our result shows that L-LPS induced shearing of DNA in mouse macrophages confirming the production of ROS (figure 7*d*). Altogether, our results indicate that L-LPS induces both TNF-α- and NO-mediated apoptosis.

## 3. Discussion

*Leptospira* has several pathogenic serovars infecting a variety of hosts leading to diverse infections which may be asymptomatic, chronic or acute [29]. This may be attributed to the difference in serovar-specific activation of the innate immune response. LPS is a major antigen eliciting both innate and adaptive immune responses and contributing to virulence [30]. Since LPS defines the serovar specificity and may have a significant contribution in the outcome of infection in a different host, it was our interest to test the ability of LPS isolated from different pathogenic serovars in inducing a differential innate response in macrophages from both resistant (mouse) and susceptible (human and bovine) hosts. Considering a huge number of serovars (greater than 300), it would be technically difficult and practically impossible to immunocharacterize all of them; hence we chose LPS from important pathogenic serovars of *Leptospira interrogans* having varied host susceptibility and prevalent in Indian scenario including LPS from non-pathogenic *Leptospira biflexa*. Our result shows that L-LPS obtained from different serovars induced differential response as they were more proinflammatory in mouse and less stimulatory in human and bovine macrophages, and overall less potent than E-LPS. Moreover, there was also the difference in LPS isolated from different serovars in their ability to induce an inflammatory response in macrophages (figure 2*a*,*b*). Although bacterial LPS is generally considered a highly conserved molecule, previous studies have shown that LPS from different bacterial species have different potency or ability to activate innate immune cells. For instance, LPS from *P. gingavalis* has been shown to be less potent than *E. coli* LPS [31]. A recent study on biochemical analysis of LPS from different pathogenic and non-pathogenic *Leptospira* serovars revealed significant structural differences that might contribute to differential activation of innate response [26]. Bonhomme *et* *al.* have shown that lipoproteins may get co-purified with LPS and hence may have a significant contribution to its innate immune activity [32]. We used a modified phenol–chloroform purification method to get highly pure LPS without any significant contamination of protein or DNA (figure 1). As expected, the isolated LPS from all four serovars showed a significantly lower level of endotoxicity than *E. coli* LPS (figure 1*d*). Hence, this lower endotoxicity of L-LPS might be correlated to its overall lower level of innate activity than E-LPS.

LPS is a typical TLR4 ligand; however, L-LPS is atypical and known to activate human macrophages through differential recognition of both TLR2 and TLR4 [24,33]. Since TLR2 and TLR4 play important roles in protection against leptospirosis and this differential recognition was attributed to co-purified lipoprotein, it was of interest to test the innate activity of our highly pure LPS in different host macrophages [34,35]. Our result shows that similar to E-LPS, L-LPS activates mouse macrophages through TLR4. Interestingly, it activates macrophages from a susceptible human host through TLR2, which is consistent with a previous study (figure 3). Owing to a lack of reagents, we could not confirm this in bovine macrophages; however, our result clearly indicates that there is differential recognition of L-LPS by macrophages of susceptible and resistant hosts. Additionally, this TLR4 recognition of L-LPS was independent of CD14 (a membrane protein involved in transferring LPS molecules), further confirming the absence of any lipoprotein or LPS binding protein (figure 3*b*). Although we have not done structural characterization, it is likely that our purified LPS preparation is of full length as a previous study has shown that shorter LPS from *L*. *interrogans* Manilae strains M895 binds strongly to CD14, whereas full-length LPS from strain L495 has a lower affinity [32]. Our observation is also in accordance with a previous study that described poor binding of CD14 to atypical LPS of *Helicobacter pylori* and *P. gingivalis* [36].

TLR4 signalling can be mediated through both MyD88 and TRIF adapter, and our results show that similar to E-LPS, L-LPS mediates signalling primarily through MyD88 except P-LPS which induced dual signalling involving both MyD88 and TRIF (figure 4*a*,*b*). The activation of both MyD88-dependent and -independent (TRIF-mediated) pathways by P-LPS may be attributed to some of the structural differences which determine initial binding to TLR and MD2 and subsequent conformational changes creating more or less binding sites for recruitment of different adaptor proteins (MyD88 or TRIF) [37]. Previous studies have shown that LPS from different bacterial species can enhance the activation of either TLR4-MyD88-dependent or -independent pathways. For instance, *Salmonella* LPS enhanced activation of the TLR4-MyD88-independent pathway, whereas the MyD88-dependent pathway was dominant in cells stimulated with *E. coli* or *V. cholerae* LPS [38]. It has been shown that upon LPS stimulation, NF-kb translocation is primarily dependent on MyD88; however, TRIF is required for TNF-α transcription [39]. The mitogen-activated protein kinase (MAPK) signalling pathway involving mediators like P38, ERK and JNK plays a vital role in regulating expression levels of inflammatory cytokines [40,41]. Differential contributions of these pathways to inflammatory responses of the activated macrophage signalling impact the dynamics of NF-κB translocation via ERK1/2, or JNK, or P38 [42]. Our result shows that in contrast to *E.coli* LPS, which induces activation via JNK and ERK, L-LPS induced activation via P38 and ERK (figure 4*d*). ERK is known to be activated upon LPS stimulation of different cell types [43]. Previous studies have shown that LPS from different bacterial species, like *P. gingivalis* and *E. coli,* use different MAPK signalling pathways to induce proinflammatory responses in human monocytes [44]. Since P38 MAPK regulates the production of IL-12, whereas the ERK1/2 pathway modulates the production of IL-10, the ability of L-LPS to produce both regulatory cytokines indicates that it might play an important role in coordinated induction of both Th1 and Th2 response [45].

LPS is known to be a potent apoptotic agent and can induce early apoptosis by the autocrine TNF-α secretion or late apoptosis by the production of NO, predominantly seen in macrophages [21]. Previous studies have shown that *Leptospira* can induce apoptosis, and LPS does have a role in this process by inducing the expression and cytomembrane translocation of Fas/FasL proteins through MAPK signalling pathways [46]. However, it was of interest to check if there is any difference in apoptotic potency of LPS from different serovars across different species macrophages, and our result shows that L-LPS induced both early and late apoptosis and LPS isolated from non-pathogenic serovar was more potent than those isolated from pathogenic serovars. Since the deleterious effects of LPS are associated with the secretion of TNF-α, and NO predominantly by tissue macrophages, significantly lower level of both TNF-α and NO induced by L-LPS in our study further confirms its lower endotoxicity than E-LPS.

*Leptospira* can activate NLRP3 inflammasome in mouse macrophages via signalling through its LPS, lipoproteins and glycoproteins [47]. A recent study has demonstrated the role of *Leptospira* LPS in activating both canonical and non-canonical inflammasome [25]. Our results demonstrate that LPS from all serovars were able to activate canonical inflammasome and induce IL-1β production (figure 6*a*). Further, in contrast to E-LPS, L-LPS did not induce cleavage of GSMD leading to a low level of IL-1β and subsequent pyroptosis which is consistent with a previous study [25]. Since *Leptospira* enters and egresses from macrophages without getting killed, LPS recognition and subsequent activation of the non-canonical inflammasome might play a significant role in the intracellular survival of bacteria [48]. Considering the fact that non-canonical inflammasome is usually activated by intracellular LPS, we delivered the purified LPS inside macrophages and tested its activation. L-LPS activated non-canonical inflammasome with an expression of Cas11 and induction of IL-1β, which is consistent with a previous study [25]. However, in a previous study by Bonhomme *et al.* [25], they did not prime the macrophages with PAM3CSK4, which is essential for the generation of pro-caspases. Moreover, the amount of LPS used for transfection was very low (500 ng), whereas we used 2–5 µg, which is a standard amount used in several studies [49,50].

In conclusion, our study has demonstrated that LPS isolated from three important pathogenic serovars induces a differential innate response in various host cells. Further, L-LPS differs from E-LPS in several aspects. Being atypical, it is less endotoxic, induces signalling through TLR2 in human macrophages, activates inflammasome without pyroptotic-induced cell death, and also induces a lesser degree of apoptosis. Thus overall, less potency of L-LPS than E-LPS may be attributed to structural differences; however, this needs to be explored. Our study provides a clear insight into the immunomodulatory role of *Leptospira* LPS and shows that the response against this antigen may determine the outcome of the infection in a particular host.

## 4. Material and methods

### 4.1. Cell lines and reagents

A mouse macrophage cell line (RAW264.7), a human embryonic kidney cell line (HEK293) and a human monocytic cell line (THP1) were initially purchased from the American Type Culture Collection (Manassas, VA). Mouse macrophage wild-type (WT; NR-9456), TLR2 KO (NR-9457), TLR4 KO (NR-9458), TLR2&4 DKO (NR-19975), TRIF KO (NR-9566), MyD88 KO (NR-15633), MyD88 and TRIF DKO (NR-15632), and CD14 KO (NR-9570) cell lines were obtained from BEI Resources, USA. BoMac cells were a kind gift from Dr Judy Stabel (Johne's Disease Research Project, USDA-ARS-NADC). RAW264.7 and knock-out mouse macrophage cells were cultured in DMEM, whereas THP1 and BoMac cells were cultured in RPMI-1640 (Sigma, USA) supplemented with 10% FBS (Invitrogen, Carlsbad, CA, USA), penicillin (100 U ml^−1^) and streptomycin (100 mg ml^−1^) and maintained at 37°C in a humidified incubator (5% CO_2_). PAM3CSK4 (TLR-2 ligand) was procured from Invitrogen, whereas *E. coli* LPS (0111: B4) was procured from Sigma Aldrich. Inhibitors of NF-kB (SN50), P38 (SB203580), MEK1 and MEK2 inhibitor (U0126), and JNK (SP600125) were procured from Invivogen. Mouse, human and bovine cytokine estimation kits were from R&D Biosystems. PerCP-CY5.5-conjugated anti-mouse MHC-II, APC-conjugated anti-mouse CD80, PE-conjugated anti-mouse CD86, BV421-conjugated anti-mouse CD40, BV421-conjugated anti-human HLR, PerCp-Cy5.5-conjugated anti-human CD80, BV650-conjugated anti-human CD86, antibodies for flow cytometry experiments, were procured from BD Biosciences, USA. Bovine-specific FITC-conjugated CD80, PE-conjugated CD86 and FITC-conjugated MHC-II antibodies for flow cytometry were procured from Invitrogen, USA. All reagents were purchased from Sigma unless otherwise specified.

### 4.2. Animals

Male C57BL/6 mice (five to six weeks) were obtained from the Animal Resource and Experimental Facility of the National Institute of Animal Biotechnology (NIAB), Hyderabad. Mice were kept under standard pathogen-free conditions and received water and food ad libitum at the facility. All the animal procedures were performed according to the Institutional Animal Ethics Committee (IAEC) norms, regulated by the Committee for the Purpose of Control and Supervision of Experiments on Animals (CPCSEA) of India.

### 4.3. Bacterial strains and culture

Pathogenic *Leptospira interrogans* serovar Icterohaemorragiae strain RGA, serovar Pomona strain Pomona, serovar Hardjoprajitno strain Hardjo and non-pathogenic *Leptospira biflexa* serovar semeranga strain Patoc1 were obtained from *Leptospira* Reference Unit, National Institute of Epidemiology, Chennai, India. Four strains of *Leptospira* were cultivated at 28°C in Ellinghausen-McCullough-Johnson-Harris (EMJH) medium supplemented with 10% EMJH Enrichment (BD, Maryland).

### 4.4. LPS isolation and purification

LPS from all four serovars were isolated by hot water/phenol extraction method using the published procedure [51]. Briefly, bacterial cells were harvested from mid-log cultures by centrifugation at 10 000 r.p.m. for 15 min and then washed three times with PBS having 150 mM CaCl_2_ and 500 mM MgCl_2_. The cell pellet was resuspended in PBS with MgCl_2_ and CaCl_2_ and sonicated to rupture the cells, treated with DNase-I and RNase-A, followed by Proteinase-K (100 µg ml^−1^) digestion as per the manufacturer's specifications. The aqueous phase was extensively dialysed against Milli-Q water (six changes for three days) to remove the phenol and then lyophilized. Stock solutions of 1 mg ml^−1^ of purified lyophilized LPS were prepared using endotoxin-free water (Millipore). Purified LPS were visualized by modified periodic acid staining following the published method [52]. The protein and nucleic acid contamination was removed by treating the samples with Proteinase-K, RNase-A and DNase-I and then confirmed by loading 30 µg of L-LPS boiled in the 5X Lamellae buffer on 12% SDS-PAGE and 2% agarose gels, respectively, and then visualized on Gel Doc.

### 4.5. Endotoxin activity assay

The endotoxin levels in LPS preparation were assessed using a Pierce LAL Chromogenic Endotoxin Quantitation Kit, as per the manufacturer's recommendations. LPS were thoroughly vortexed, sonicated and then tested at varying concentrations (0.1 µg ml^−1^, 1 µg ml^−1^, 10 µg ml^−1^ and 20 µg ml^−1^) in duplicates using the kit.

### 4.6. Isolation and propagation of bone marrow-derived dendritic cells

Bone marrow-derived dendritic cells were prepared as described previously [53]. Briefly, bone marrow recovered from the femur and tibia of mice was passed through a 70 µm cell strainer in a sterile culture dish containing a complete DMEM. The cell suspension was centrifuged at 1000 r.p.m. for 5 min, and the supernatant was discarded. The RBCs were lysed by ACK lysis buffer, washed trice and suspended in a complete medium. 1 × 10^7^ bone marrow cells per well were cultured in 6-well plates in 4 ml of complete DMEM supplemented with GM-CSF (20 ng ml^−1^) and IL-4 (5 ng ml^−1^) (Peprotech). On days 2 and 7, half of the medium was replaced with a new medium supplemented with GM-CSF (40 ng ml^−1^) and IL-4 (10 ng ml^−1^). On day 9, non-adherent and loosely adherent cells were removed. The adherent cells were then harvested by gentle washing with PBS, pooled and used for specific assays.

### 4.7. Cytokine estimation

Sandwich ELISA kits (R&D Systems) were used to measure cytokine levels in culture supernatant in various experiments, following the manufacturer's instructions. In experiments designed to determine the optimal LPS dose, RAW264.7 or THP1 or BoMac cells were seeded onto 24-well plates, followed by stimulation with 500 ng ml^−1^ of E-LPS and various concentrations (0.25/0.5/1/2/5/10 µg ml^−1^) of L-LPS purified from the four serovars. In a separate experiment, WT, TLR2 KO, TLR4 KO and TLR2&4 DKO, TRIF KO, MyD88 KO, MyD88&TRIF DKO, CD14 KO cell lines were stimulated with PAM3CSK4 (20 ng ml^−1^) or E-LPS (500 ng ml^−1^) or L-LPS (2 µg ml^−1^) for 24 h at 37°C/5% CO_2_ and cytokines (IL-6, TNF-α, IL-1β, IL-12, IL-10, IL-23) were measured in the culture supernatant by sandwich ELISA kit. To access the signalling pathway involved, additional experiments were done in which RAW264.7 cells were pre-treated for 30 min at 37°C with inhibitors to NF-kB (520 µM), JNK (40 µM), MEK1 and MEK2 inhibitor (25 µM) or P38 (230 µM) and then stimulated with L-LPS for 24 h at 37°C, and cytokines (IL-6, TNF-α) were measured in culture supernatant by sandwich ELISA kit. The human monocyte (THP1) cells were stimulated with PMA (5 ng ml^−1^) treatment for 48 h to get macrophages and rested further for 48 h to reduce the effect of PMA stimulation. Human monocyte-derived macrophages and BoMac cells were seeded into the 24-well format and unstimulated or stimulated with E-LPS (500 ng ml^−1^) or L-LPS (0.025–10 µg ml^−1^) for 24 h, and the supernatant was used to estimate the levels of cytokines using the sandwich ELISA kits.

### 4.8. Flow cytometric analysis

Mouse, human and bovine macrophage cells were incubated in 24-well plates (10^6^ cells per well) with PAM3CSK4 (20 ng ml^−1^), E-LPS (500 ng ml^−1^) or L-LPS (2 µg ml^−1^) for 24 h. Cells were harvested, washed with prechilled PBS, blocked with blocking solution (0.5% BSA and 2% FBS in PBS) for 30 min followed by three washes and then stained with PerCp-Cy5.5-conjugated anti-mouse MHC-II, APC-conjugated anti-mouse CD80, PE-conjugated anti-mouse CD86, and CD40, BV421-conjugated anti-human HLR-DR, PerCp Cy5.5-conjugated anti-human CD80, BV650-conjugated anti-human CD86, FITC-conjugated anti-bovine CD80, PE-conjugated anti-bovine CD86, and FITC-conjugated anti-bovine MHC-II antibodies for 1 h on ice. The cells were washed trice, fixed with 1% paraformaldehyde, and then 100 000 events were acquired using a BD-LSR Fortesa and analysed using FlowJo.

### 4.9. Nitric oxide estimation

Nitric oxide (NO) production in L-LPS-stimulated macrophages was determined in the culture supernatant by measuring the stable end-products of NO using the method of Miranda *et al*. [54]. Briefly, 100 µl of vanadium chloride and 50 µl of Griess reagent were added to 100 µl culture supernatant and incubated at 37°C for 40 min. The absorbance was measured at 550 nm, and the nitrite concentration (nitric oxide indicator) was calculated from the standard curve generated from serial dilutions of NaNO_2_ (3.12–200 µM in DMEM).

### 4.10. qRT-PCR

RAW264.7 or THP1 or BoMac cell lines were seeded into a 6-well plate and treated with L-LPS (2 µg ml^−1^) or E-LPS (500 ng ml^−1^) for 4 h or 24 h. After treatment, cells were recovered, and RNA was purified using RNAeasy Kit (MN) following the manufacturer's protocol. First-strand cDNA was synthesized using a superscript III-RT system (Invitrogen), and two-step amplification was performed in a 10 µl reaction volume containing 50 ng cDNA, 5pM each primer, and SYBR green (Takara). Samples were run in triplicate, and data were analysed with Sequence Detection System (BIO-RAD). The experimental data were presented as fold changes of gene expression of stimulated cells at various time points relative to the control. mRNA levels of the analysed genes were normalized to the amount of b-actin or GAPDH present in each sample. Primers were synthesized by Bioserva and their sequences are given in electronic supplementary material, table S1.

### 4.11. Confocal microscopy

Leptospires were labelled with CFSE following the published protocol with minor modifications [55]. Briefly, *Leptospira* was washed twice in DPBS to remove the proteins in the EMJH media and diluted in 1 ml of DPBS containing 5 µM CFSE and then incubated for 30 min at room temperature, protected from light. The excess CFSE was washed with DPBS supplemented with 1% BSA, and bacteria were counted using a counting chamber under a microscope. RAW264.7 cells were grown overnight on coverslips and then stimulated with E-LPS (500 ng ml^−1^) or L-LPS (2 µg ml^−1^) for 6 h at 37°C in 5% CO_2_ in complete DMEM without antibiotics. The cells were washed with DPBS and infected with CFSE-labelled *Leptospira* at an MOI of 1 : 20 for 15 min in a CO_2_ incubator. The cells were then washed extensively 10 times with DPBS and then fixed for 15 min using 4% paraformaldehyde. Whereas for phagocytosis assay, LPS-stimulated macrophages were infected with CFSE-labelled *Leptospira* at an MOI of 1 : 100 for 90 min, and cells were washed with DMEM and then treated for 1 h with DMEM containing gentamycin (30 µg ml^−1^) to kill the extracellular *Leptospira*. The cells were fixed in permeabilization buffer (4% formaldehyde with 0.5% TritonX-100 in PBS) for 15 min, followed by three washes with PBS. Cells were blocked for 1 h in PBS containing 5% (w/v) BSA and then stained with rabbit–anti mouse-EEA1 (1 : 200, CST; 3288S) for 1 h in PBS containing 1% BSA and 0.05% saponin. After five quick washes, the cells were incubated with 2° antibody Alexa Fluor 555 conjugated anti-rabbit IgG (1 : 500, CST; 4413) for 30 min, washed thrice with DPBS, mounted with VECTA SHIELD (containing DAPI) and observed using a ×63 oil objective on a confocal microscope (Leica SP8, Wetzlar, Germany). ‘Z-Stack images’ were taken at different cross-sections of the macrophages perpendicularly to the bottom of the slide.

### 4.12. Western blot analysis

RAW264.7 cells were stimulated with E-LPS (500 ng ml^−1^) or L-LPS (5 µg ml^−1^) for 24 h at 37°C/5% CO_2_. For analysing the non-canonical inflammasome, RAW264.7 cells were primed with PAM3CSK4 (20 ng ml^−1^) for 2 h, followed by transfection with E-LPS and L-LPS for 5 h. These treated cells were recovered and lysed with RIPA cell lysis buffer supplemented with proteinase and phosphatase inhibitor mixture (Sigma Aldrich). The lysate was then centrifuged at 4°C, 12 000*g*, for 30 min, and protein concentrations in the supernatant were measured using the BCA protein-quantification assay (ChemCruz). Equal amounts of protein were loaded, separated by SDS-PAGE and then transferred electrophoretically to PVDF membranes (0.22 µm, Millipore, Bedford, MA, USA). After blocking with 5% BSA in Tris-buffered saline (TBS; pH 7.4), the membranes were incubated overnight at 4°C with primary Abs, anti-P38 (1 : 1000); anti-phospho-P38, anti-ERK anti-phospho-ERK, anti-stress-activated protein kinase/JNK, anti-phospho-stress-activated protein kinase/JNK, anti-ASC (antibodies were procured from Santa Cruz Biotechnology, Beverly, MA, USA); anti-capase-11, anti-caspase-1, anti-GSMD and HRP-conjugated β-actin procured from Cell Signalling Technology (CST), with dilution according to the manufacturer's instructions. After three washes with TBS-T (0.05% (v/v) Tween-20), the membranes were incubated for 1–2 h at room temperature with the corresponding HRP-conjugated anti-rabbit/anti-mouse/anti-rat IgG secondary Ab (1 : 5000, CST, USA) diluted in TBS. The housekeeping gene (b-actin) expression controls loading an equal amount of total protein concentration. The peroxidase-positive bands were detected using an ECL detection kit.

### 4.13. Annexin-V assay

Annexin-V interacts strongly (specifically) with phosphatidyl serine to detect early apoptosis by targeting the loss of plasma membrane asymmetry. RAW264.7 or THP1 or BoMac cells were stimulated with actinomycin D (ActD : positive control; 100 ng ml^−1^) or E-LPS (1 µg ml^−1^) or L-LPS (2 µg ml^−1^) for 6 h in a CO_2_ incubator. The attached cells were then washed with PBS, detached using Accutase (Sigma Aldrich), and combined with the supernatant. 20 µl of 5× Annexin-V binding buffer (100 mM HEPES, 40 mM KCl, 1 M NaCl, 7.5 mM MgCl_2_, 25 mM CaCl_2_, pH 7.4) with Annexin-V–Alexa488 and propidium iodide (PI; 20 µM ml^−1^) was added and incubated at room temperature for 15 min. Excess and unbounded antibodies were removed by washing the cells three times in washing buffer (0.5% (w/v) BSA and 0.05% (w/v) sodium azide in PBS). Cells were fixed by 1% PFA and analysed with a BD Fortessa flow cytometer. A complete analysis was done using FlowJo, a three-star software.

### 4.14. Reactive oxygen species measurement

Mitochondrial reactive oxygen species (ROS) were measured by treating the RAW264.7 cells grown on 12-well format or coverslips with E-LPS (500 ng ml^−1^) or L-LPS (2 µg ml^−1^), followed by staining with MitoSOX red, following the manufacturer's instructions. Briefly, post-induction, the cells were washed with DPBS three times and then incubated with MitoSOX red for 30 min at 37°C, washed trice in DPBS, and fixed with 4% paraformaldehyde in PBS (Chemcruz, USA) for 15 min at RT and washed four times with PBS. Cells were mounted onto slides using mounting media containing DAPI (Vectashield, Vector Labs) and sealed using nail polish. The slides were analysed by confocal microscopy (MitoSOX red; excitation wavelength 555/28; emission wavelength 617/73), observed using a ×63 oil objective on a confocal microscope (Leica SP8, Wetzlar, Germany). Whereas for the flow cytometry analysis, cells were detached from the culture plate using Accutase treatment for 5 min at 37°C, followed by inhibiting the enzymatic activity by adding FBS. The cells were washed with FACS buffer (DPBS with 0.5% BSA and 2% FBS) three times and incubated with MitoSOX red dye for 30 min at 37°C, followed by three washes with wash buffer. The cells were pelleted down and resuspended in 1% paraformaldehyde in PBS (ChemCruz, USA) and acquired 100 000 events per sample with BD LSR-Fortessa flow cytometer with PE (585/42) filter. A complete analysis was done using FlowJo, a three-star software.

### 4.15. Analysis of apoptotic bodies

DNA fragmentation due to inter-nucleosome cleavage was determined as reported previously [56]. Briefly, 5 × 10^6^ macrophages were treated with ActD (100 ng ml^−1^) or E-LPS (2 µg ml^−1^), or L-LPS(5 µg ml^−1^) for 24 h and then harvested and washed in ice-cold PBS. The cells were lysed in 0.5 ml of lysis buffer (50 mM l^−1^ Tris–HCl, 10 mM l^−1^ EDTA, 1% SDS, pH 8.0) for 16 h at 4°C, and the lysates were centrifuged (15 000*g* for 20 min at 4°C) to separate high-molecular-weight DNA (pellet) from cleaved low-molecular-weight DNA (supernatant). The DNA supernatants were phenol-extracted twice, and ethanol (95%) precipitated overnight at 4°C for 16 h. The samples were pelleted down and resuspended in Tris-EDTA buffer containing 250 µg ml^−1^ Rnase-A (Sigma Aldrich). The samples were heated at 65°C for 10 min and subjected to electrophoresis in a 2% agarose gel containing ethidium bromide.

### 4.16. Quantification and statistical analysis

The figure captions mention all statistical tests, comparisons among the groups and the number of repetitions. All data are represented as the mean ± s.d. Error bars indicate standard deviations. All other statistical analyses were performed using one-way or two-way analysis of variance (ANOVA). Significant differences among the means of the groups were determined by multiple comparison analyses using the statistical hypothesis of the Dunnett and Sidak test for two-way and one-way ANOVA, respectively. In all analyses: ^#^*p* < 0.0001, ***p < 0.001, ∗∗*p* < 0.01, ∗*p* < 0.05, n.s. = not statistically significant (*p* > 0.05). The experiments were replicated at least three times, and all attempts at replication were successful. All statistical analyses were performed using GraphPad (Prism 8).

## Data Availability

All data generated or analysed during this study are included in this article. Further enquiries can be directed to the corresponding author. All data are provided in the figures and electronic supplementary material [57].

## References

[1] Adler B, Faine S. 1977 Host immunological mechanisms in the resistance of mice to leptospiral infections. Infect. Immun. **17**, 67-72. (10.1128/iai.17.1.67-72.1977) 885617 PMC421082

[2] Costa F, Wunder Jr EA, De Oliveira D, Bisht V, Rodrigues G, Reis MG, Ko AI, Begon M, Childs JE. 2015 Patterns in Leptospira shedding in Norway rats (*Rattus norvegicus*) from Brazilian slum communities at high risk of disease transmission. PLoS Negl. Trop. Dis. **9**, e0003819. (10.1371/journal.pntd.0003819) 26047009 PMC4457861

[3] Goarant C. 2016 Leptospirosis: risk factors and management challenges in developing countries. Res. Rep. Trop. Med. **7**, 49-62. (10.2147/RRTM.S102543) 30050339 PMC6028063

[4] Grassmann AA, Souza JD, McBride AJ. 2017 A universal vaccine against leptospirosis: are we going in the right direction? Front Immunol. **8**, 256. (10.3389/fimmu.2017.00256) 28337203 PMC5343615

[5] Medzhitov R, Janeway Jr C. 2000 Innate immunity. N. Engl. J. Med. **343**, 338-344. (10.1056/NEJM200008033430506) 10922424

[6] Pasare C, Medzhitov R. 2005 Control of B-cell responses by Toll-like receptors. Nature **438**, 364-368. (10.1038/nature04267) 16292312

[7] Wille-Reece U et al. 2006 Toll-like receptor agonists influence the magnitude and quality of memory T cell responses after prime-boost immunization in nonhuman primates. J. Exp. Med. **203**, 1249-1258. (10.1084/jem.20052433) 16636134 PMC2121207

[8] Kelley N, Jeltema D, Duan Y, He Y. 2019 The NLRP3 inflammasome: an overview of mechanisms of activation and regulation. Int. J. Mol. Sci. **20**, 3328. (10.3390/ijms20133328) 31284572 PMC6651423

[9] Martinon F, Burns K, Tschopp J. 2002 The inflammasome: a molecular platform triggering activation of inflammatory caspases and processing of proIL-beta. Mol. Cell. **10**, 417-426. (10.1016/s1097-2765(02)00599-3) 12191486

[10] Wang Z, Zhang S, Xiao Y, Zhang W, Wu S, Qin T, Yue Y, Qian W, Li L. 2020 NLRP3 inflammasome and inflammatory diseases. Oxid. Med. Cell Longev. **2020**, 4063562. (10.1155/2020/4063562) 32148650 PMC7049400

[11] Huang X et al. 2019 Caspase-11, a specific sensor for intracellular lipopolysaccharide recognition, mediates the non-canonical inflammatory pathway of pyroptosis. Cell Biosci. **9**, 31. (10.1186/s13578-019-0292-0) 30962873 PMC6438033

[12] Kayagaki N et al. 2013 Noncanonical inflammasome activation by intracellular LPS independent of TLR4. Science **341**, 1246-1249. (10.1126/science.1240248) 23887873

[13] Shi J, Zhao Y, Wang Y, Gao W, Ding J, Li P, Hu L, Shao F. 2014 Inflammatory caspases are innate immune receptors for intracellular LPS. Nature **514**, 187-192. (10.1038/nature13683) 25119034

[14] Liu X, Zhang Z, Ruan J, Pan Y, Magupalli VG, Wu H, Lieberman J. 2016 Inflammasome-activated gasdermin D causes pyroptosis by forming membrane pores. Nature **535**, 153-158. (10.1038/nature18629) 27383986 PMC5539988

[15] Alexander C, Rietschel ET. 2001 Bacterial lipopolysaccharides and innate immunity. J. Endotoxin. Res. **7**, 167-202. 11581570

[16] Park BS, Lee JO. 2013 Recognition of lipopolysaccharide pattern by TLR4 complexes. Exp. Mol. Med. **45**, e66. (10.1038/emm.2013.97) 24310172 PMC3880462

[17] Ciesielska A, Matyjek M, Kwiatkowska K. 2021 TLR4 and CD14 trafficking and its influence on LPS-induced pro-inflammatory signaling. Cell Mol. Life Sci. **78**, 1233-1261. (10.1007/s00018-020-03656-y) 33057840 PMC7904555

[18] Islam MA, Uddin MJ, Tholen E, Tesfaye D, Looft C, Schellander K, Cinar MU. 2013 Age-associated differential production of IFN-gamma, IL-10 and GM-CSF by porcine alveolar macrophages in response to lipopolysaccharide. Vet. J. **198**, 245-251. (10.1016/j.tvjl.2013.07.026) 23985297

[19] Wu TT, Chen TL, Chen RM. 2009 Lipopolysaccharide triggers macrophage activation of inflammatory cytokine expression, chemotaxis, phagocytosis, and oxidative ability via a toll-like receptor 4-dependent pathway: validated by RNA interference. Toxicol. Lett. **191**, 195-202. (10.1016/j.toxlet.2009.08.025) 19735705

[20] Yang Y, Wang H, Kouadir M, Song H, Shi F. 2019 Recent advances in the mechanisms of NLRP3 inflammasome activation and its inhibitors. Cell Death Dis. **10**, 128. (10.1038/s41419-019-1413-8) 30755589 PMC6372664

[21] Xaus J, Comalada M, Valledor AF, Lloberas J, Lopez-Soriano F, Argiles JM, Bogdan C, Celada A. 2000 LPS induces apoptosis in macrophages mostly through the autocrine production of TNF-alpha. Blood **95**, 3823-3831. (10.1182/blood.V95.12.3823) 10845916

[22] Murray GL, Srikram A, Henry R, Hartskeerl RA, Sermswan RW, Adler B. 2010 Mutations affecting *Leptospira interrogans* lipopolysaccharide attenuate virulence. Mol. Microbiol. **78**, 701-709. (10.1111/j.1365-2958.2010.07360.x) 20807198

[23] Bulach DM, Kalambaheti T, de la Pena-Moctezuma A, Adler B. 2000 Lipopolysaccharide biosynthesis in Leptospira. J. Mol. Microbiol. Biotechnol. **2**, 375-380. 11075908

[24] Nahori MA, Fournie-Amazouz E, Que-Gewirth NS, Balloy V, Chignard M, Raetz CR, Saint Girons I, Werts C. 2005 Differential TLR recognition of leptospiral lipid A and lipopolysaccharide in murine and human cells. J. Immunol. **175**, 6022-6031. (10.4049/jimmunol.175.9.6022) 16237097

[25] Bonhomme D, Hernandez-Trejo V, Papadopoulos S, Pigache R, Fanton d'Andon M, Outlioua A, Boneca IG, Werts C. 2023 *Leptospira interrogans* prevents macrophage cell death and pyroptotic IL-1β release through its atypical lipopolysaccharide. J. Immunol. **210**, 459-474. (10.4049/jimmunol.2200584) 36602965

[26] Vanithamani S et al. 2021 Biochemical analysis of leptospiral LPS explained the difference between pathogenic and non-pathogenic serogroups. Microb. Pathog. **152**, 104738. (10.1016/j.micpath.2021.104738) 33529737

[27] Li S et al. 2018 *Leptospira interrogans* infection leads to IL-1β and IL-18 secretion from a human macrophage cell line through reactive oxygen species and cathepsin B mediated-NLRP3 inflammasome activation. Microb. Infect. **20**, 254-260. (10.1016/j.micinf.2018.01.010) 29432801

[28] Zheng D, Liwinski T, Elinav E. 2020 Inflammasome activation and regulation: toward a better understanding of complex mechanisms. Cell Discov. **6**, 36. (10.1038/s41421-020-0167-x) 32550001 PMC7280307

[29] Ko AI, Goarant C, Picardeau M. 2009 Leptospira: the dawn of the molecular genetics era for an emerging zoonotic pathogen. Nat. Rev. Microbiol. **7**, 736-747. (10.1038/nrmicro2208) 19756012 PMC3384523

[30] Needham BD, Carroll SM, Giles DK, Georgiou G, Whiteley M, Trent MS. 2013 Modulating the innate immune response by combinatorial engineering of endotoxin. Proc. Natl Acad. Sci. USA **110**, 1464-1469. (10.1073/pnas.1218080110) 23297218 PMC3557076

[31] Diya Z, Lili C, Shenglai L, Zhiyuan G, Jie Y. 2008 Lipopolysaccharide (LPS) of *Porphyromonas gingivalis* induces IL-1β, TNF-α and IL-6 production by THP-1 cells in a way different from that of *Escherichia coli* LPS. Innate Immun. **14**, 99-107. (10.1177/1753425907088244) 18713726

[32] Bonhomme D, Santecchia I, Vernel-Pauillac F, Caroff M, Germon P, Murray G, Adler B, Boneca IG, Werts C. 2020 Leptospiral LPS escapes mouse TLR4 internalization and TRIFassociated antimicrobial responses through O antigen and associated lipoproteins. PLoS Pathog. **16**, e1008639. (10.1371/journal.ppat.1008639) 32790743 PMC7447051

[33] Werts C et al. 2001 Leptospiral lipopolysaccharide activates cells through a TLR2-dependent mechanism. Nat. Immunol. **2**, 346-352. (10.1038/86354) 11276206

[34] Chassin C et al. 2009 TLR4- and TLR2-mediated B cell responses control the clearance of the bacterial pathogen, *Leptospira interrogans*. J. Immunol. **183**, 2669-2677. (10.4049/jimmunol.0900506) 19635914

[35] Viriyakosol S, Matthias MA, Swancutt MA, Kirkland TN, Vinetz JM. 2006 Toll-like receptor 4 protects against lethal *Leptospira interrogans* serovar icterohaemorrhagiae infection and contributes to *in vivo* control of leptospiral burden. Infect. Immun. **74**, 887-895. (10.1128/IAI.74.2.887-895.2006) 16428731 PMC1360355

[36] Cunningham MD, Seachord C, Ratcliffe K, Bainbridge B, Aruffo A, Darveau RP. 1996 *Helicobacter pylori* and *Porphyromonas gingivalis* lipopolysaccharides are poorly transferred to recombinant soluble CD14. Infect. Immun. **64**, 3601-3608. (10.1128/iai.64.9.3601-3608.1996) 8751905 PMC174269

[37] Hoebe K, Janssen EM, Kim SO, Alexopoulou L, Flavell RA, Han J, Beutler B. 2003 Upregulation of costimulatory molecules induced by lipopolysaccharide and double-stranded RNA occurs by Trif-dependent and Trif-independent pathways. Nat. Immunol. **4**, 1223-1229. (10.1038/ni1010) 14625548

[38] Zughaier SM, Zimmer SM, Datta A, Carlson RW, Stephens DS. 2005 Differential induction of the toll-like receptor 4-MyD88-dependent and -independent signaling pathways by endotoxins. Infect. Immun. **73**, 2940-2950. (10.1128/IAI.73.5.2940-2950.2005) 15845500 PMC1087371

[39] Sakai J, Akkoyunlu M. 2017 The role of BAFF system molecules in host response to pathogens. Clin. Microbiol. Rev. **30**, 991-1014. (10.1128/CMR.00046-17) 28855265 PMC5608883

[40] Liu Y, Shepherd EG, Nelin LD. 2007 MAPK phosphatases—regulating the immune response. Nat. Rev. Immunol. **7**, 202-212. (10.1038/nri2035) 17318231

[41] Schoenberg DR, Maquat LE. 2012 Regulation of cytoplasmic mRNA decay. Nat. Rev. Genet. **13**, 246-259. (10.1038/nrg3160) 22392217 PMC3351101

[42] Liu T, Zhang L, Joo D, Sun SC. 2017 NF-κB signaling in inflammation. Signal Transduct. Target. Ther. **2**, 17023. (10.1038/sigtrans.2017.23) 29158945 PMC5661633

[43] Beinke S, Robinson MJ, Hugunin M, Ley SC. 2004 Lipopolysaccharide activation of the TPL-2/MEK/extracellular signal-regulated kinase mitogen-activated protein kinase cascade is regulated by IκB kinase-induced proteolysis of NF-κB1 p105. Mol. Cell. Biol. **24**, 9658-9667. (10.1128/MCB.24.21.9658-9667.2004) 15485931 PMC522219

[44] Yiemwattana I, Chaisomboon N, Yeesibsan J, Pongcharoen S. 2017 Differential induction of MAPK signaling pathways by *Porphyromonas gingivalis* and *Escherichia coli* lipopolysaccharide in human monocytes. J. Int. Dental Med. Res. **10**, 1-5. (10.7727/wimj.2016.081)

[45] Arthur JS, Ley SC. 2013 Mitogen-activated protein kinases in innate immunity. Nat. Rev. Immunol. **13**, 679-692. (10.1038/nri3495) 23954936

[46] Du P, Li SJ, Ojcius DM, Li KX, Hu WL, Lin X, Sun AH, Yan J. 2018 A novel Fas-binding outer membrane protein and lipopolysaccharide of *Leptospira interrogans* induce macrophage apoptosis through the Fas/FasL-caspase-8/−3 pathway. Emerg. Microbes Infect. **7**, 135. (10.1038/s41426-018-0135-9) 30061622 PMC6066479

[47] Lacroix-Lamande S, d'Andon MF, Michel E, Ratet G, Philpott DJ, Girardin SE, Boneca IG, Vandewalle A, Werts C. 2012 Downregulation of the Na/K-ATPase pump by leptospiral glycolipoprotein activates the NLRP3 inflammasome. J. Immunol. **188**, 2805-2814. (10.4049/jimmunol.1101987) 22323544

[48] Paciello I et al. 2013 Intracellular Shigella remodels its LPS to dampen the innate immune recognition and evade inflammasome activation. Proc. Natl Acad. Sci. USA **110**, E4345-E4354. (10.1073/pnas.1303641110) 24167293 PMC3832022

[49] Lagrange B et al. 2018 Human caspase-4 detects tetra-acylated LPS and cytosolic Francisella and functions differently from murine caspase-11. Nat. Commun. **9**, 242. (10.1038/s41467-017-02682-y) 29339744 PMC5770465

[50] Ye J et al. 2021 Scutellarin inhibits caspase-11 activation and pyroptosis in macrophages via regulating PKA signaling. Acta Pharm. Sin. B **11**, 112-126. (10.1016/j.apsb.2020.07.014) 33532184 PMC7838020

[51] WHO. 2011 Report of the Second Meeting of the Leptospirosis Burden Epidemiology.

[52] Fomsgaard A, Freudenberg MA, Galanos C. 1990 Modification of the silver staining technique to detect lipopolysaccharide in polyacrylamide gels. J. Clin. Microbiol. **28**, 2627-2631. (10.1128/jcm.28.12.2627-2631.1990) 1704012 PMC268246

[53] Faisal SM, Chen JW, McDonough SP, Chang CF, Teng CH, Chang YF. 2011 Immunostimulatory and antigen delivery properties of liposomes made up of total polar lipids from non-pathogenic bacteria leads to efficient induction of both innate and adaptive immune responses. Vaccine **29**, 2381-2391. (10.1016/j.vaccine.2011.01.110) 21300103

[54] Miranda KM, Espey MG, Wink DA. 2001 A rapid, simple spectrophotometric method for simultaneous detection of nitrate and nitrite. Nitric Oxide **5**, 62-71. (10.1006/niox.2000.0319) 11178938

[55] Santecchia I, Bonhomme D, Papadopoulos S, Escoll P, Giraud-Gatineau A, Moya-Nilges M, Vernel-Pauillac F, Boneca IG, Werts C. 2022 Alive pathogenic and saprophytic leptospires enter and exit human and mouse macrophages with no intracellular replication. Front. Cell Infect. Microbiol. **12**, 936931. (10.3389/fcimb.2022.936931) 35899053 PMC9310662

[56] Hogquist KA, Nett MA, Unanue ER, Chaplin DD. 1991 Interleukin 1 is processed and released during apoptosis. Proc. Natl Acad. Sci. USA **88**, 8485-8489. (10.1073/pnas.88.19.8485) 1924307 PMC52533

[57] Varma VP, Bankala R, Kumar A, Gawai S, Faisal SM. 2023 Differential modulation of innate immune response by lipopolysaccharide of *Leptospira*. Figshare. (10.6084/m9.figshare.c.6856630) PMC10645091 37935355

